# Progress in the application of hyperspectral imaging technology in quality detection and in the modernization of Chinese herbal medicines

**DOI:** 10.3389/fchem.2025.1620154

**Published:** 2025-06-20

**Authors:** Yuting You, Lei Zhang, Zhuo Yu, Daqing Zhao, Xueyuan Bai, Wei Zhang

**Affiliations:** ^1^ Research Center of Traditional Chinese Medicine, The Affiliated Hospital to Changchun University of Chinese Medicine, Changchun, China; ^2^ Northeast Asia Research Institute of Traditional Chinese Medicine, Changchun University of Chinese Medicine, Changchun, China

**Keywords:** hyperspectral imaging, non-destructive, Chinese herbal medicine, quality evaluation, classification and identification

## Abstract

Hyperspectral imaging (HSI) technology integrates spectral analysis and image recognition with non-destructive and efficient advantages, and is widely used in the agriculture, geological exploration, military sectors, among others. Traditional Chinese medicine (TCM) has a long history of use in China, and to ensure the quality of TCM herbs, it is necessary to perform accurate quality assessments. It is also crucial to evaluate the active ingredients and changes in cultivation strategies and processing parameters over time. The use of HSI technology for the investigation of Chinese medicines has grown in importance, and recent advances in HSI have enabled the multi-dimensional non-destructive analyses of various components, origins, and growth statuses, thereby providing innovative solutions for modernization. This paper systematically reviews the application of HSI for detecting active ingredients, evaluating their quality, and recognizing the authenticity and species of Chinese herbal medicines. It clearly describes the limitations of hyperspectral technology in terms of data processing, emphasizes the importance of textural information, and suggests the application of HSI for large-scale detection.

## 1 Introduction

Traditional Chinese medicine (TCM) refers to natural medicines used under the guidance of traditional Chinese medicine theory. These medicines are primarily derived from plants, animals, and minerals, and are processed into medicinal products to regulate bodily functions, in addition to preventing, treating, and diagnosing various diseases. The earliest use of TCM in China can be traced back to ancient times. More specifically, during the Xia, Shang, and Zhou periods, its development began to be documented through systematic theoretical research. Ancient methods for identifying the quality of TCM herbs mainly relied on the accumulation of experience and sensory judgment, and combined with the technical conditions and theories of Chinese medicine at that time, a complete set of identification methods were gradually formed, including sensory identification, fire, and water assays. However, these methods are highly subjective, are unable to accurately quantify Chinese herbal medicines, and are incompatible with processed products.

With the development of modern science and technology, researchers have devoted themselves to the in-depth analysis and study of TCM herbs using the novel techniques that have become available to them over the years. As a result, new quality control methods, including microscopic identification, chemical identification, and stable isotope technologies, have been proposed and applied. For example, compared with naked-eye observation microscopic identification is based on the use of microscopy to observe the cellular structures of herbs and provide more intuitive information. However, its operation is complicated, especially when the herbs have been processed, since the resulting structural changes may complicate their identification (Zhang Hui et al., 2024). Alternatively, chemical identification methods, including high-performance liquid chromatography and gas chromatography, represent highly sensitive techniques, and can provide more objective and reliable data; however, some limitations remain in terms of separating and identifying complex components, thereby rendering it difficult to fully assess interactions and synergistic effects (Wang et al., 2022). Stable isotope techniques, on the other hand, have been applied to identify the geographic origins of herbs. The ratios of common stable isotopes (e.g., carbon, hydrogen, and oxygen) present in the herbs originating from different regions and subjected to different growth environments can vary significantly, thereby allowing traceability evaluation be performed (Yu et al., 2022). However, these methods are destructive and are unsuitable for rare or intact materials. Additionally, previous studies assessed only individual samples, failing to meet the requirements of holistic quality evaluations. Therefore, the combination of multiple methods for the comprehensive assessment of herbs will be an important trend in the future quality control of TCM ingredients (Indrayanto, 2024).

**FIGURE 1:**
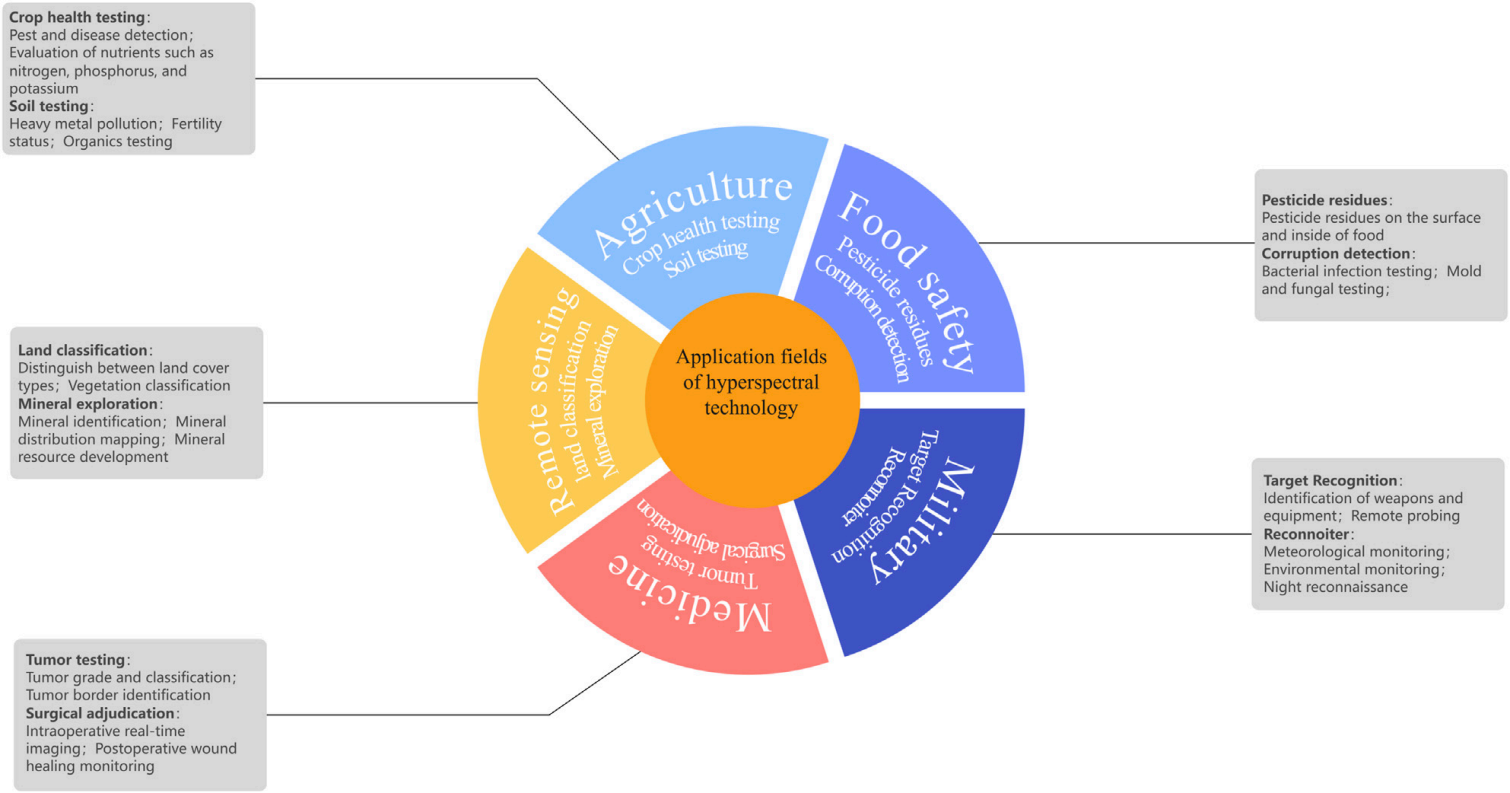
Applications of hyperspectral imaging technology in various fields.

Hyperspectral imaging (HSI) is an imaging technique that acquires and analyzes the spectral information of target objects across multiple continuous narrow bands within the visible and near-infrared spectra to identify their chemical compositions and physical properties. As a general concept, spectroscopy has its origins in the early 20th century, and since then has been commonly employed in the fields of astronomy, physics, and chemistry, with a focus on substance identification and analysis using spectral data (Figure 1). Although early technologies focused on limited spectral bands, they paved the way for the development of HSI. In the 1960s, with the rise of remote sensing, multispectral imaging gained prominence, while in 1987, the development of the first HSI system marked a significant breakthrough. By the 21st century, advances in sensors and computational power had boosted its capabilities. Moreover, due to its notable advantages, such as its high sensitivity, rapid nature, and non-invasive operation, HSI is now widely used across multiple fields, especially in the fields of agriculture (Liu et al., 2020; Cheshkova, 2022), food science (Lv et al., 2023), defense (Luft et al., 2014), geology (Chakraborty et al., 2024), medicine (Sharma et al., 2024) and cultural relics protection (Shi et al., 2024). As a result, this technology has led to the development of novel methods for the quality control and compositional analysis of Chinese herbal medicines, boosting the efficiency of research, while providing reliable quality control support. As related technologies continue to advance and improve, their potential applications in traditional Chinese medicine continue to expand.

With new developments in the area of HSI, the spectral resolution has been increased down to the pixel level, with each pixel containing spectral information across multiple bands. This allows the creation of a spectral “curve” that reflects the absorption, reflection, and scattering characteristics of the target object at different wavelengths. To date, various data processing techniques have been employed to analyze spectral signatures and to distinguish and identify various substances, thereby enabling detailed feature recognition and quantitative analysis of the types, qualities, and components of Chinese herbal medicines (Zhang et al., 2021). Hyperspectral technology not only captures wavelength information, but it also records the two-dimensional spatial information of objects to generate a three-dimensional data cube. In contrast to traditional data, high-dimensional data provide richer information. Through the subsequent analyses of these spectral data using deep learning algorithms, the characteristic differences in medicinal materials and the distribution of bioactive compounds within them can be visually presented, ultimately advancing the standardization and scientific progress of traditional Chinese medicine, and addressing the limitations associated with traditional identification methods (Zhang Deng-Ting et al., 2023; Wang He et al., 2023).

However, relying solely on spectral information often fails to capture the full characteristics of such materials. For example, textural information, which represents a key metric for describing local image regions and patterns, reflects the distributions, arrangements, and variations in pixel intensities. Thus, a combination of both spectral and textural information provides a fuller picture of the surface structure and shape. By analyzing grayscale differences between bands and pixel directional features, it is possible to supplement spatial structure details and provide more complete data for medicinal herb identification and quality assessment. Moreover, the effective fusing of spectral and textural information not only significantly improves the detection accuracy and reliability, but it also opens new research possibilities in other fields (Wang et al., 2020; Duan et al., 2024). For example, in agriculture, textural information aids in the detection of diseased areas, contributes to analysis of the soil quality, and can be used to distinguish between different crop types (Zhao et al., 2023). In the food industry, textural information provides details related to surface defects, helps detect food spoilage, and contributes to the identification of food adulteration (Wang Yulong et al., 2024). Moreover, in medicine, textural information aids in disease diagnosis and pathological analysis, including in the detection of skin conditions and tumors, and in the assessment of lesion types and progression degrees (Colomer et al., 2020). These examples clearly demonstrate that the integration of spectral and textural information through the use of HSI technology not only guides quality control in medicinal herbs, but that it also promotes material identification and improves the classification accuracy across multiple fields.

However, as far as we know, in the field of traditional Chinese medicine, the systematic and comprehensive description of monitoring technology and data fusion is still insufficient. This article reviews the application of HSI in the identification of the origin of traditional Chinese medicine, classification of varieties, quality assessment and planting monitoring, etc. Furthermore, this paper also focuses on discussing the key role of texture information in feature data recognition, and systematically sorts out the fusion methods of spectral information and texture information at multiple scales. Meanwhile, the article reviews the commonly used preprocessing methods, feature extraction methods and modeling strategies for different types of data. The core objective of this study is to provide relevant researchers with an overall research trend analysis of the application of HSI technology in the field of traditional Chinese medicine, thereby promoting the effective transformation of monitoring technology from theoretical research to practical application.

## 2 Overview of HSI technology

### 2.1 Acquisition of HSI data

Acquiring hyperspectral data is a key step in HSI technology, wherein an imaging spectrometer is used to collect spectral information from target objects. Depending on the imaging principles, hyperspectral instruments can be classified into two types, namely, push brooms and snapshots. In line-scanning HSI using a push-broom approach, the sensor scans the entire scene line by line from one direction, representing an efficient technique for large-area imaging. In area-scanning HSI using a snapshot approach, all scene data are obtained simultaneously, which is ideal for scanning dynamic objects.

**FIGURE 2:**
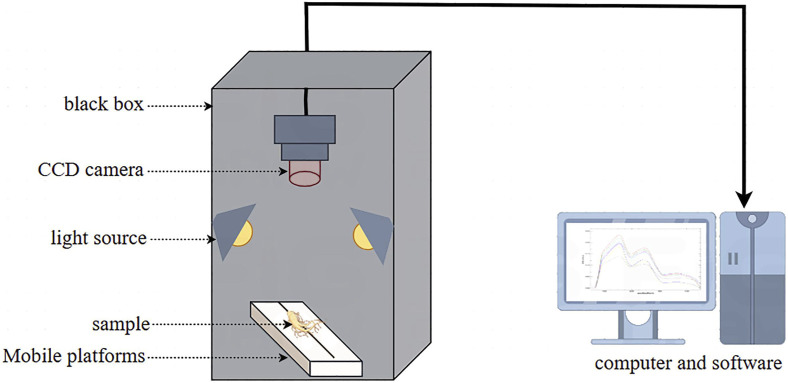
Hyperspectral imaging system.

Spectral data acquisition involves three steps, including scene selection, equipment calibration, and data collection. Following the selection of an observation scene based on the application requirements, the equipment (see Figure 2) is calibrated in terms of both spectral and geometric calibrations. For spectral calibration, standard panels, such as whiteboards, are used to remove the effects of ambient light, while geometric calibration ensures that the positional features of the image match those of the actual ground objects. Finally, the parameters for data collection include the light sources, methods, wavelength ranges, exposure times, sampling frequencies, and resolutions.

### 2.2 Methods for the processing of spectral information

Because hyperspectral data encompass the chemical compositions and physical structures of materials, they are characterized by large data volumes, high degrees of dimensionality, and significant noise. Data processing therefore aims to extract key information from a vast dataset to permit the identification, classification, or quantitative analysis of different substances. The processing steps include preprocessing, dimensionality reduction, spectral feature extraction, classification, and recognition. More specifically, data preprocessing aims to reduce or eliminate noise and acquisition artifacts, as well as normalizing the data scale across different samples and spectral bands to facilitate subsequent analysis. Common methods include normalization and smoothing filtration. In addition, dimensionality reduction involves reducing the data dimensionality or increasing the class separability using mathematical methods. Techniques associated with this processing step include principal component analysis, independent component analysis, linear discriminant analysis, and factor analysis, all of which contribute to simplifying the data structure. Indeed, this represents the most critical step in spectral data processing, as it involves selecting the most representative features from the raw data. Examples include calculating the band ratios to obtain vegetation indices (Table 1), or analyzing the continuum features of spectral curves to identify material compositions and structures. Classification and recognition represent the ultimate objectives of hyperspectral data processing, and can be typically categorized into supervised classification, unsupervised classification, and deep learning algorithms. For supervised classification using labeled sample data, various algorithms can be employed, including support vector machines, decision trees, and random forests. Unsupervised classification groups data into categories based on intrinsic similarities, often employing clustering algorithms such as k-means clustering and hierarchical clustering, which are multilayer neural networks comprising input, output, and hidden layers. They can automatically extract features from the data and learn complex nonlinear relationships. Common algorithms include feed-forward neural networks, convolutional neural networks (CNNs), and long short-term memory networks.

**TABLE 1:** Commonly used vegetation indices and formulae.

No.	Vegetation index	Abbreviation	Formula	References
1	Chlorophyll Absorption Ratio Index	CARI	$\left(\left|a*R_{670}+R_{670}+b\right|*R_{700}\right)/\left[\text{SQRT}\left(a^{2}+1\right)*R_{670}\right]$ $a=\left(R_{700}-R_{550}\right)/150,b=R_{550}-500*a$	Kim et al. (1994)
2	Modified Chlorophyll Absorption in Reflectance Index	MCARI	$\left[\left(R_{700}-R_{670}\right)-0.2\left(R_{700}-R_{550}\right)\right]\left(R_{700}/R_{670}\right)$	Daughtry et al. (2000)
3	Transformed Chlorophyll Absorption Reflectance Index	TCARI	$3*\left[\left(R_{700}-R_{670}\right)-0.2*\left(R_{700}-R_{550}\right)*\left(R_{700}/R_{670}\right)\right]$	Haboudane et al. (2002)
4	Renormalized Difference Vegetation Index	RDVI	$\left(R_{800}-R_{670}\right)/\text{SQRT}\left(R_{800}-R_{670}\right)$	Roujean and Breon, (1995)
5	Photochemical Reflectance Index	PRI	$R_{531}-R_{570}/R_{531}+R_{570}$	Gamon, Penuelas, and Field, (1992)
6	Green Normalized Difference Vegetation Index	GNDVI	$R_{750}-R_{540}-R_{570}/R_{750}+R_{540}+R_{570}$	Gitelson and Merzlyak, (1997)
7	Normalized difference Vegetation Index	NDVI	$R_{800}-R_{670}/R_{800}+R_{670}$	Rouse Jr et al. (1974)
8	Optimised Soil Adjusted Vegetation Index	OSAVI	$\left(1+0.16\right)\left(R_{800}-R_{670}\right)/\left(R_{800}+R_{670}+1.16\right)$	Rondeaux, Steven, and Baret, (1996)
9	Structure Insensitive Pigment Index	SIPI	$\left(R_{800}-R_{445}\right)/\left(R_{800}+R_{430}\right)$	Penuelas, Baret, and Filella, (1995)
10	Double Peak Index	DPI	$\left(R_{688}*R_{710}\right)/{R_{697}}^{2}$	Zarco-Tejada et al. (2003)
11	Triangular Vegetation Index	TVI	$120\left(R_{750}-R_{550}\right)-200\left(R_{670}-R_{550}\right)/2$	Broge and Leblanc, (2001)
12	Normalized Pigment Chlorophyll Index	NPCI	$\left(R_{680}-R_{430}\right)/\left(R_{680}+R_{430}\right)$	Vigier, Pattey, and Strachan, (2004)
13	Plant Senescence Reflectance Index	PSRI	$\left(R_{660}-R_{510}\right)/R_{760}$	Merzlyak et al. (1999)
14	MERIS Terrestrial Chlorophyll Index	MTCI	$\left(R_{754}-R_{709}\right)/\left(R_{709}+R_{681}\right)$	Dash and Paul (2004)
15	Normalized Difference Red Edge Index	NDRE	$\left(R_{790}-R_{720}\right)/\left(R_{790}+R_{720}\right)$	Barnes et al. (2000)
16	Anthocyanin Reflectance Index	ARI	$R_{700}/R_{550}-1$	Gitelson (2004)
17	Plant Pigment Ratio	PPR	$\left(R_{550}-R_{450}\right)/\left(R_{550}+R_{450}\right)$	Metternicht, (2003)
18	Modified Simple Ratio	MSR	$\left(R_{800}/R_{670}-1\right)/\sqrt{R_{800}/R_{670}+1}$	Chen, (1996)
19	Normalized Pheophytization Index	NPQI	$\left(R_{415}-R_{430}\right)/\left(R_{415}+R_{430}\right)$	Barnes et al. (1992)

### 2.3 Methods for the extraction of textural information

Various methods exist for extracting textural features, and these can be primarily categorized into statistical analysis, structural analysis, signal processing, and model-based approaches (Table 2). Statistical methods include the gray-level co-occurrence matrix and local binary patterns. The former calculates the gray-level and positional relationships between pixel pairs to describe textural features, such as the contrast and energy, whereas the latter compares adjacent pixel gray levels using binary encoding to build a histogram for textural analysis (Alibabaei et al., 2023). Structural analysis primarily uses a histogram of oriented gradients, which divides an image into cells, computes the gradient direction distributions within each cell, groups cells into blocks for normalization, and concatenates these normalized histograms to detect the edges and shapes present in the objects.

**TABLE 2:** Common textural features and formulae.

No.	Textural feature	Formula
1	Mean	$\sum_{i=1}^{N}\sum_{j=1}^{N}i*P\left(i,j\right)$ 1
2	Variance	$\sum_{i=1}^{N}\sum_{j=1}^{N}P\left(i,j\right)\left(i-\text{Mean}\right)$ 1
3	Standard deviation	$\sqrt{\sum_{i=1}^{N}\sum_{j=1}^{N}P\left(i,j\right){\left(i-\text{mean}\right)}^{2}}$ 1
4	Homogeneity (Bai et al., 2019)	$\sum_{i=1}^{N}\sum_{j=1}^{N}P\left(i,j\right)/{1+\left(i-j\right)}^{2}$ 1
5	Contrast (Akimov et al., 2019)	$\sum_{i=1}^{N}\sum_{i=1}^{N}{\left(i-j\right)}^{2}P\left(i,j\right)$ 1
6	Dissimilarity	$\sum_{i=1}^{N}\sum_{j=1}^{N}P\left(i,j\right)\left|i-j\right|$ 1
7	Entropy (Barnes et al., 1992)	- $\sum_{i=1}^{N}\sum_{j=1}^{N}P\left(i,j\right)\mathrm{lg}\left(p\left(i,j\right)\right)$ 1
8	ASM	$\sum_{i=1}^{N}\sum_{j=1}^{N}{P\left(i,j\right)}^{2}$ 1
9	Correlation (Arivazhagan et al., 2013)	$\sum_{i=1}^{N}\sum_{i=1}^{N}\left(\text{ij}\right)P\left(i,j\right)-\mu_{i}\mu_{j}/\sigma_{i}\sigma_{j}$ 1

Textures often exhibit different characteristics at varying scales because they provide multilevel information. Techniques such as the Fourier and wavelet transformations decompose information at multiple scales and convert spatial data to the frequency domain to extract textures at specific orientations and scales. In addition, model-based methods offer new approaches for extracting textural features. For example, CNNs fuse deep features to enhance complex textural recognition, and have been employed in various applications to date (Cai et al., 2022). In practice, ongoing improvements in the computing power and in the associated algorithms lead to a continuous evolution of textural extraction methods and tools. Moreover, a combination of deep features with traditional textural features has the potential to boost the accuracy and robustness of image classification (Gao et al., 2021); however, it also poses new challenges for feature analysis.

### 2.4 Method for the fusion of spectral and textural information

**FIGURE 3:**
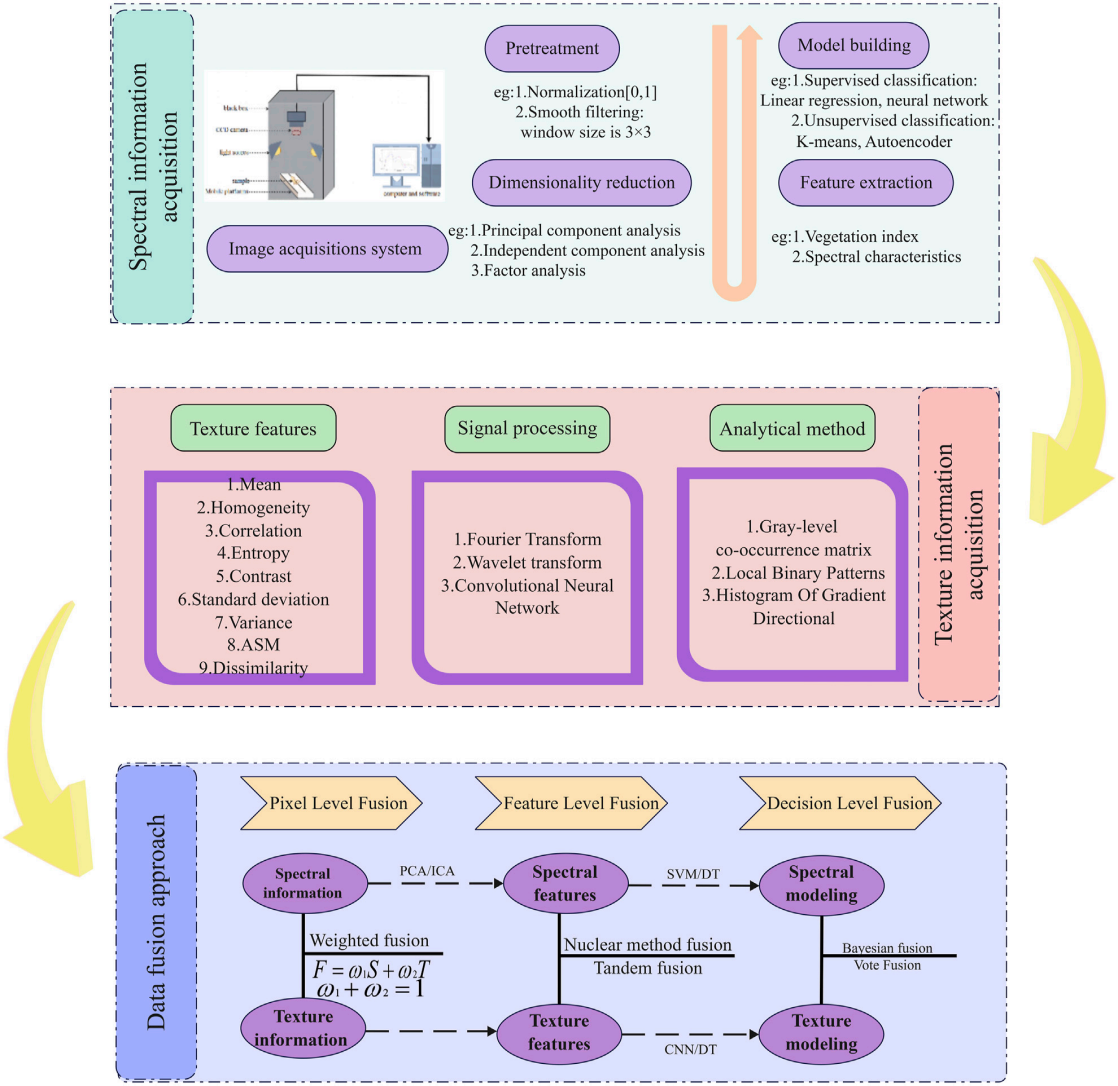
Spectral data acquisition, textural information acquisition, and the fusion approach.

The fusion of spectral and textural information is a common technique in image processing and computer vision, and has emerged as an important research direction in image analysis in recent years (Figure 3). It was therefore considered desirable to merge these two techniques to achieve more precise image classification, object detection, and feature extraction. Currently available fusion methods are based on pixel-, feature-, and decision-level fusion.

For example, pixel-based fusion methods combine two information sources directly at the pixel level, simplifying complex information and rendering its analysis more facile. For example, spectral and textural images can be layered using a weighted average method, or specific frequency band features can be fused using filter methods. Although pixel-based fusion methods effectively enhance the image contrast, they also introduce artifacts and edge blurring, which render it difficult to balance information differences at different scales in some complex application scenarios. Consequently, such approaches are only suitable for processing high-resolution image data. As a result, feature- and decision-level fusion methods offer several benefits for many applications. For example, feature-level fusion methods pull features from various information sources and then merge these features at specific levels to form a new, more comprehensive feature vector, effectively enhancing the classification accuracy (Zhou et al., 2022a). Previous research in this area has shown that combining spectral features with wavelet texture features can significantly enhance the accuracy of remote sensing estimates of rice leaf area indices based on data collected from unmanned aerial vehicles (i.e., drones). However, the contributions of individual features can differ between applications, thereby rendering selection of the best features rather challenging. Additionally, following direct combination, strong correlations between features can interfere with the final results. To address this, decision-based fusion methods can be employed, which initially handle the classification or detection of different information sources separately, and then integrate the results at the decision level to generate the final decision. Decision-level fusion occurs primarily at the output layer of the classifiers, which can reduce the chances of mistakes from a single classifier to enhance the overall robustness of the system. In addition, compared with the first two methods, decision-level fusion does not require complex data processing, instead simply merging the results of each classifier, and rendering the integration of multiple data sources more straightforward. However, decision-level fusion has lower information usage and relies excessively on classifier performance. Thus, the performance of the classifiers is low, the results will not be ideal. The above points clearly demonstrate that each of the three fusion methods exhibits specific advantages and disadvantages. Choosing the appropriate fusion method therefore requires careful consideration of the data characteristics, application goals, and real-time computing requirements, as well as balancing the advantages and disadvantages of each method, and using appropriate optimization techniques to achieve the optimal fusion effect.

## 3 Application of HSI in the compositional analysis of TCM

HSI is an advanced technology for material analysis, which is based on spectral information and has been widely used in the compositional analysis of TCM in recent years. Using this approach, researchers have performed various quantitative analyses, and have studied the spatial distributions of the components present in traditional Chinese medicine by collecting reflectance or transmittance spectral data across various wavelengths. Additionally, with the ongoing development of artificial intelligence and machine learning technology, HSI technology has shown good application prospects in the quality control, composition identification, and traceability analysis of Chinese herbal medicines (Pan et al., 2024; Yi et al., 2020).

### 3.1 Quality assessment of TCM

#### 3.1.1 Establishment and validation of quality standards

In the application of hyperspectral technology, the establishment and validation of quality control standards for TCM materials has recently become an important area of focus in the field of traditional Chinese medicine quality testing. For example, following the determination of characteristic spectral fingerprints using spectral data and data mining technologies, conducted correlation analysis was performed based on the chemical and pharmacodynamic components to establish quality standards for TCM. As previously reported, HSI technology can effectively distinguish between Chinese herbal medicines of different origins, thereby supporting the development of quality standards (Yang et al., 2020). Furthermore, relevant studies have indicated that hyperspectral technology exhibits a high sensitivity and specificity for the quality detection of Chinese herbal medicines, effectively identifying pollutants and counterfeit products (Xu et al., 2023). To verify the accuracy and reliability of HSI technology in the quality assessment of Chinese herbal medicines, it is necessary to compare the safety and pharmacodynamic verification data with those of traditional quality control methods. In addition, textural information should be coupled with physical characteristics (e.g., color and structure) to ensure the diversity of indicators during the establishment of quality standards.

In the application of compositional analysis and authenticity identification, hyperspectral data collection refers to the collection of data from a single Chinese herbal medicine. However, quality standards require the collection of data from numerous samples of different types and origins, and which have been subjected to different processing methods. Following data processing and modeling, specific testing standards and processes are formulated based on national or industry standards, and these are subsequently combined with actual testing requirements to ensure their consistency and reproducibility under different conditions (Christophe et al., 2005). Overall, the establishment of quality standards for TCMs based on hyperspectral technology not only leads to improved scientific quality control, but it also systematically integrates the germplasm resources of Chinese herbal medicines, establishes a sharing platform, and promotes the multifaceted collaboration of quality standards for Chinese herbal medicines.

#### 3.1.2 Quantitative analysis of active ingredients

The application of hyperspectral technology has gradually become a research hotspot for the quantitative analysis of TCM components. Through the use of HSI technology, it is possible to obtain the spectral features of Chinese herbal medicine components across multiple subgroups, whilst also incorporating the use of machine learning algorithms. This establishes a quantitative relationship between the spectral features and the chemical composition, and permits a quantitative analysis of the active ingredients present in the TCM. As reported previously, the active ingredients present in TCMs (e.g., flavonoids, saponins, and anthraquinones) exhibit unique absorption and reflection features in different bands of the hyperspectrum, which can be used to further estimate the concentration and distribution of these components (He et al., 2018; Shi et al., 2022). In addition, the positions and intensities of the absorption peaks within the different wavelength ranges may also differ, thereby allowing effective identification of the corresponding chemical components. Furthermore, previous works have determined the number of growth years of kudzu roots using this property in combination with deep learning algorithms. Furthermore, with the advancement of related technologies and the cross-application of multiple disciplines, the implementation of HSI technology in quantitative analysis is expected to become more widespread. Consequently, it should be possible to comprehensively assess the quality of TCM and ensure the stability and validity of its components (Lin et al., 2021; Shen et al., 2024), thereby providing new ideas and methods for the standardization and quality control of Chinese herbal medicines.

#### 3.1.3 Research progress in multi-component synergistic analysis

The application of hyperspectral technology for the multicomponent synergistic analysis of Chinese herbal medicines is progressing rapidly. By simultaneously acquiring rich spectral information, hyperspectral technology can not only be used for the quantitative detection and synergistic effect analysis of multiple active ingredients in such products, but it can also be employed to obtain information regarding the component distributions in different parts these herbal medicines through spatial analysis technology. Consequently, the spatial distribution characteristics of the biologically active ingredients can be revealed, thereby highlighting the application potential of hyperspectral technology in the field of traditional Chinese medicine. Hyperspectral technology has also been used to collect spectra from different parts of ginseng, and the partial least squares and Principal Component Analysis (PCA) models have been combined to clarify the distribution characteristics of ginsenosides and other components (Zhang W. et al., 2024). In addition, flavonoids are the main components of the traditional Chinese medicine Scutellaria baicalensis, and by combining hyperspectral technology with machine learning algorithms, it is possible to simultaneously analyze the distributions of flavonoids, apricots, and other components in Scutellaria baicalensis. Furthermore, spectral and pharmacological data have been combined to clarify the distributions of polysaccharides, flavonoids, and carotenoids in Lycium barbarum, which has provided an objective basis for exploring the active ingredients of this shrub in terms of their antioxidant and immunomodulatory properties (Zhang et al., 2020). Moreover, using pixel-level recognition technology, the distributions of trace components have been detected in Chinese herbal medicines, including trace mycotoxins in red ginseng, thereby providing an important basis for safety assessments (Liu Biao et al., 2024). It is therefore evident that hyperspectral technology shows strong potential for use in the spatial analysis of component distributions in Chinese herbal medicines, and could provide new technical support for quality evaluations and medicinal efficacy assessments of such products (Table 3).

**TABLE 3:** Examples of hyperspectral imaging techniques for compositional analysis.

No.	Varieties employed for content prediction	Performance parameters	References
1	Purple Potato	Spectral range: 900–1700 nm Spectral resolution: 5 nm Spectral bands:256	Heo et al. (2021)
2	Winter Jujube	Spectral range: 400–1000 nm Exposure time: 28 ms Spectral bands:128	Wei et al. (2024a)
3	Dried Ginger	Spectral range: 400–1000 nm Spectral resolution: 7 nm Spectral bands:204	Samrat et al. (2022)
4	Atractylodis Rhizoma	Spectral range: 400–1000 nm 900–1700 nm Spectral resolution: 8 nm Spectral bands:512	Jiang et al. (2023b)
5	Mulberry Fruits	Spectral range: 40–1000 nm Spectral resolution: 2.8 nm Exposure time: 60 ms	Li et al. (2023a)
6	Flos Lonicerae	Spectral range: 400–1000 nm Spectral resolution: 1.58 nm Exposure time: 90 ms	Liu et al. (2019)
7	Turmeric	Spectral range: 400–1000 nm Spectral resolution: 2.3 nm	Farrar et al. (2021)
8	Glycyrrhiza	Spectral range: 500–900 nm Spectral resolution:9 nm Spectral bands:45	Xu et al. (2024)
9	Milk	Spectral range: 400–1000 nm Spectral resolution: 4.8 nm Spectral bands:125	Zhang and Liu, (2025)
10	Chrysanthemum	Spectral range: 900–1700 nm	He et al. (2021)
11	Coix Seeds	Spectral range: 491–2500 nm Spectral bands:396	Wang et al. (2023b)
12	Lycium	Spectral range: 900–1700 nm Spectral resolution: 5 nm Spectral bands:256	Zhang et al. (2020)
13	Ganoderma	Spectral range: 400–1000 nm 900–1700 nm	Ran et al. (2025)
14	Gastrodia	Spectral range: 400–2500 nm	Ma et al. (2023)
15	Jujube	Spectral range: 900–1700 nm Spectral resolution: 5.13 nm	Ibrahim et al. (2021)
16	Orange peel	Spectral range: 900–2500 nm	Badaró et al. (2020)
17	Honey	Spectral range: 400–1000 nm Spectral bands:128	Lanjewar, Panchbhai, and Patle, (2024)
18	Raw yams	Spectral range: 900–1700 nm Spectral resolution: 8 nm	Adesokan et al. (2024)
19	Lotus Seed	Spectral range: 380–1030 nm Spectral resolution: 2.8 nm	Wei et al. (2023)
20	Panax notoginseng powder	Spectral range: 400–1000 nm Spectral resolution: 2.8 nm	Sun et al. (2024)
21	Flos Lonicerae	Spectral range: 371–1024 nm Spectral resolution: 2.8 nm	Wang et al. (2019b)
22	Loquat	Spectral range: 400–1000 nm Spectral resolution: 2.8 nm Spectral bands:360	Li et al. (2023b)
23	Puerariae Thomsonii Radix	Spectral range: 900–2500 nm Spectral bands:288	Hu et al. (2023)
24	Wolfberry	Spectral range:400–1000 nm Spectral resolution: 2.53 nm Spectral bands:256	Chen et al. (2024)
25	Pomelo peel	Spectral range: 1000–2500 nm Spectral resolution:8 nm	Chen et al. (2019)
26	Blueberry	Spectral range: 900–1700 nm Spectral bands:512	Qiu et al. (2024b)
27	Hetian jujube	Spectral range: 1000–2500 nm Spectral resolution: 5.43 nm	Wei et al. (2024b)

### 3.2 Identification of Chinese herbal medicines

The identification of Chinese herbal medicines is an important link in the research and application of TCMs, and is known to involve many aspects, such as quality control, the evaluation of medicinal effects, and the clinical application of herbs. With the rapid development of the Chinese medicine industry, there is an increasing demand for the identification of Chinese herbal medicines, especially to ensure their authenticity, efficacy, and safety. Previous studies have shown that the combination of hyperspectral technology with deep learning algorithms can effectively identify different types of Chinese herbal medicines, thereby providing a greater analytical accuracy and significantly reducing detection times (Wang Qi et al., 2023). At present, the application of hyperspectral technology in the identification of Chinese herbal medicines is mainly reflected in the identification of different varieties, along with confirmation of their authenticity and quality.

#### 3.2.1 Identification of different Chinese herbal medicine varieties

Significant differences exist in the chemical compositions and structures of different Chinese herbal medicine varieties. Consequently, hyperspectral technology has been successfully applied to classify and identify different varieties of Chinese herbal medicines based on their absorption, reflection, and scattering properties at different wavelengths, along with the fusion analyses of their spectral and textural information using mathematical algorithms (Zhou et al., 2020). For example, organic compounds such as flavonoids and terpenoids have unique fingerprint characteristics in the near-infrared region. By analyzing the hyperspectral information of chrysanthemums using a deep CNN algorithm, a differentiation accuracy of ∼100% was obtained for seven varieties of chrysanthemums (Wu et al., 2018). In addition, using this technique, superior results were obtained for the species identification of different Bayberry (Kabir et al., 2022), Jujube (Liu Hong et al., 2024), Cannabis sativa (Lu et al., 2022), Potato (Li Sihai et al., 2024), Lycium barbarum (Tang et al., 2021), and Mullein (Yang et al., 2022) varieties.

#### 3.2.2 Identification of the authenticity and quality of Chinese herbal medicines

The safety and effectiveness of the clinical application of Chinese herbal medicines can be detrimentally affected by product adulteration through the incorporation of low-quality substances or the inclusion of substances with similar appearances but different compositions. Previous studies have shown that hyperspectral technology can significantly improve the efficiency and accuracy of the detection of counterfeit Chinese herbal medicines (Yi et al., 2020). More specifically, through a comprehensive analysis of multi-dimensional data based on the spectral “fingerprints” of Chinese herbal medicines containing polysaccharides, flavonoids, and saponins, the observation of different spectral responses at specific wavelengths can allow rapid identification of the product authenticity. In this context, the authentication and rapid assessment of ginseng and other valuable Chinese herbal medicines have been performed to prevent the inflow of counterfeit and inferior-quality products into the market (Wang et al., 2023c). For example, authentic wolfberries are distinguished from adulterated products based on their spectral reflectance differences in the near-infrared band (Zhang Yao et al., 2024; Nirere et al., 2023). Similarly, by establishing spectral databases of different Ganoderma samples and confusing fungal features, authentic and fake Ganoderma specimens can be effectively distinguished from one another. Moreover, in combination with deep learning algorithms, hyperspectral technology can realize a comprehensive assessment of the quality of Chinese herbal medicines, allowing rapid determination of the active ingredients to enhance quality control (Ding et al., 2024).

### 3.3 Cultivation monitoring and management of Chinese herbal medicines

With the continual growth of the Chinese medicine industry, the market demand for Chinese herbal medicine is also increasing. As a result, cultivation monitoring and management are essential to ensuring consistent yields and product qualities, in addition to improving the competitiveness of the market. At present, the application of HSI technology in cultivation monitoring is based mainly on monitoring of the soil composition, the acidity and alkalinity, real-time plant growth patterns, and real-time pest and disease infestations.

#### 3.3.1 Hyperspectral monitoring of soil and environmental factors

Recently, hyperspectral technology has been increasingly employed in the fields of soil and environmental monitoring. Different textures of soils, such as sands, clays, and loams, exhibit different spectral reflectances due to their varying particle sizes and mineral compositions. Hyperspectral technology can be used to differentiate between differently textured soils by capturing and analyzing subtle differences in spectral reflectance. In addition, since the spectral absorption characteristics of such materials are closely related to the vibrations of the pigments and other organic matter, it is possible to estimate the contents of these components and monitor the soil over a large area. For example, previous studies have shown that hyperspectral technology can effectively identify the correlation characteristics of heavy metal contamination and soil active components, which is crucial for monitoring the cultivation environments of Chinese herbal medicines (Pechlivani et al., 2023; Wang Zhihao et al., 2024). Furthermore, the combination of hyperspectral technology with Internet of Things devices (Schmidt and Ahn, 2022) and unmanned aerial systems (Huang et al., 2024) can realize dynamic monitoring of the soil quality and temperature, in addition to accurate classification of the air quality index, and the provision of data support for the precise cultivation of Chinese herbal medicines. Moreover, by analyzing the spectral data recorded for a soil, the fertilizer program and irrigation time can be adjusted to increase the fertilizer utilization rate, improve the soil structure, reduce the use of chemical pesticides, ensure the growth and quality of Chinese herbal medicines, and guarantee an optimal raw material supply.

#### 3.3.2 Real-time detection of the crop growth status

During their distinct growth stages, the leaves and canopies of crops exhibit different chemical compositions and physical structures. Using HSI, the spectral reflectances associated with multiple bands can be calculated to analyze vegetation indices that are related to crop growth. Indeed, the relationship between various characteristic parameters and crop biochemical indices has been modeled such that the chlorophyll content, photosynthetic efficiency, and water status of the crop during the growth process could be monitored in real time, which is essential for assessing the growth health of the crop. For example, studies have shown that hyperspectral technology can effectively monitor the growth status of wheat and predict the crop yield from spectral data alone (Wu et al., 2023). In addition, the use of drones carrying hyperspectral sensors enables the rapid monitoring of large crop planting areas in the dual-band range, which can lead to the detection of growth abnormalities, pests, and diseases in a timely manner, thereby allowing appropriate management measures can be taken, as necessary (Yao et al., 2019). This non-contact monitoring method therefore improves the monitoring efficiency while incorporating all measurement parameters related to the plant growth conditions and different developmental stages (Liu et al., 2021).

### 3.4 Identification of the origin of Chinese herbal medicines

The quality of Chinese herbal medicines is affected by various factors, including the soil type, climatic conditions, and altitude, all of which directly affect crop growth and development, while also influencing the accumulation of various chemical components. Consequently, when grown in a different region, the same crop can exhibit obviously different physical characteristics or pharmacodynamic components. Although traditional quality assessment methods rely on chemical analysis and sensory testing, these methods are less efficient and do not fully reflect the internal quality differences between herbs. Compared with traditional biochemical experiments, HSI technology demonstrates the unique advantage of analyzing the quality differences between Chinese herbs from different origins. More specifically, it can be used to perform a non-destructive, rapid, and multi-dimensional quality assessment of herbs and reveal their quality differences by analyzing the spectral characteristics associated with different growing environments. In this context, some studies have shown that significant differences exist between the active ingredient contents of Chinese herbs grown under different soil conditions, which can be associated with varying nutrient compositions and pH values (Chen et al., 2020). For example, in the pH 6–7 range, honeysuckle plants exhibit the highest nutrient absorption efficiency, which is favorable for the production of their active ingredient, namely, chlorogenic acid. In addition, climatic factors, such as temperature and humidity, also affect the growth of herbal medicines. For instance, the growth of Atractylodes macrocephala requires a sufficient supply of water, wherein a moist but well-drained environment ensures normal growth its tuberous roots. (Cao et al., 2024). Indeed, in-depth investigations into the effects of different growth environments on the quality of Chinese herbal medicines not only helps optimize the planting conditions, but it also provides an important reference for product quality control.

In recent years, the applicability of hyperspectroscopy in origin classification has been demonstrated (Table 4). For example, HSI has been employed to classify the geographic origins of licorice, wherein significant differences were detected in the component distributions between the investigated locations (Zhang Hui et al., 2024). In addition, Salvia miltiorrhiza, which is widely used in the treatment of cardiovascular diseases, exhibits significant differences in its active ingredients (e.g., salvianolic acid and danshenin) depending on its growth location. The application of hyperspectral technology to analyze Salvia miltiorrhiza samples demonstrated that different origins led to significant characteristic differences in the resulting spectral features of the samples. By extracting these spectral features at specific wavelengths and combining them with chemometric modeling, the origin of Salvia divinorum could be quickly distinguished, and its active ingredient content could be accurately predicted (Dai et al., 2024). Moreover, the hyperspectral technique has been combined with PCA and a partial minimization squares regression model to detect different parts of Scutellaria baicalensis, while feature band analysis has been used to reveal the differences in the flavonoid contents depending on the sample origin (Xiao et al., 2020). Furthermore, considering the importance of the saponin content of ginseng in determining its efficacy, quantitative analysis of these components can be used to effectively distinguish between samples of different origins owing to differences in the growth environment and climate, among other conditions. Consequently, spectroscopic data and chemometrics revealed that the absorption peaks of ginseng at different wavelengths varied significantly, thereby allowing clear identification of the sample origin along with a corresponding quality assessment (Ping et al., 2024).

**TABLE 4:** Examples of hyperspectral imaging techniques for origin identification.

No.	Varieties identified	Performance parameters	References
1	Lily	Spectral range: 900–1700 nm Spectral resolution: 8 nm	Zhao et al. (2024)
2	Saffron Crocus	Spectral range: 400–1000 nm Spectral resolution:3 nm Spectral bands:204	Kiani, Yazdanpanah, and Feizy, (2023)
3	Polygonatum Cyrtonema	Spectral range: 410–990 nm 950–2500 nm Spectral resolution: 6 nm Spectral bands:396	Zhang et al. (2023b)
4	Tiegun Yam	Spectral range: 410–990 nm 950–2500 nm Spectral bands:396	Zhang et al. (2023c)
5	Poria Cocos	Spectral range: 410–990 nm 950–2500 nm	Sun et al. (2023)
6	Wolfberry	Spectral range: 400–10000 nm Spectral bands:125	Hao et al. (2022a)
7	Gardeniae Fructus	Spectral range: 410–990 nm 950–2500 nm	Zhou et al. (2022b)
8	Radix Astragali	Spectral range: 400–1000 nm 900–1700 nm	Xiao et al. (2020)
9	Angelica dahurica	Spectral range: 900–1700 nm Spectral resolution: 4 nm	Xu et al. (2019)
10	Hangbaiju	Spectral range: 400–900 nm 900–2500 nm Spectral resolution: 5 nm	Long et al. (2023)
11	Wolfberry	Spectral range: 400–1000 nm Spectral resolution:2.8 nm Spectral bands:125	Hao et al. (2022b)
12	Chrysanthemum	Spectral range: 900–1700 nm	Cai et al. (2023a)
13	Wolfberry	Spectral range: 400–1000 nm 900–1700 nm Spectral bands:384	Cui et al. (2022)
14	Gentiana	Spectral range: 400–1000 nm Spectral resolution: 8 nm	Li et al. (2024a)
15	Cinnamon	Spectral range: 900–1700 nm Spectral bands:159	Cruz-Tirado et al. (2023)
16	Hazelnut	Spectral range: 1350–2500 nm Spectral resolution: 16 nm	Torres-Cobos et al. (2025)
17	Gastrodia elata Blume	Spectral range: 400–500 nm Spectral resolution: 8 nm	Liu et al. (2024c)
18	Notoginseng	Spectral range: 30-680cm^-1^ Spectral resolution:2cm^-1^	Gu et al. (2024)
19	Specialty yam	Spectral range: 400–1000 nm Spectral resolution: 4 nm	Gao et al. (2024)
20	Tangerine peel	Spectral range: 900–1700 nm Spectral bands:228	Pan et al. (2022)
21	Saffron	Spectral range: 400–950 nm	Hashemi-Nasab Parastar, (2022)
22	Fritillaria	Spectral range: 900–1700 nm Spectral resolution: 5 nm	Kabir et al. (2022)
23	Radix	Spectral range: 400–1000 nm 900–1700 nm	Cai et al. (2023b)
24	Peach and apricot kernels	Spectral resolution: 16 nm	Kajino et al. (2021)
25	Chrysanthemum	Spectral range: 900–1700 nm Spectral resolution: 5 nm	Wu et al. (2018)
26	Ginseng	Spectral range: 900–1700 nm	Cheng et al. (2024)
27	Auricularia	Spectral resolution: 8 nm	Yang et al. (2022)
28	New Zealand honey	Spectral range: 400–1000 nm Spectral resolution: 2.8 nm	Zhang and Abdulla, (2022)
29	Wolfberry fruits	Spectral range: 400–1000 nm Spectral resolution: 4.9 nm	Nirere et al. (2023)
30	Spore powder	Spectral range: 900–1700 nm Spectral bands:512	Jiang et al. (2023a)

### 3.5 Detection in the processing of Chinese herbal medicines

Following production, the processing of Chinese herbal medicines can prolong their storage times and prevent deterioration, in addition to enhancing the efficacy of the drug and moderating its potency. Processing is also conducive to compounding and mixing depending on the desired preparation. At present, HSI technology is mainly used for quality control during processing, and for confirming the compositions of the finished products.

#### 3.5.1 Quality control during processing

During the processing of Chinese herbal medicines, quality control is important to ensure the safety and effectiveness of the final product. For example, sulfur fumigation is often employed to process herbs to change their brightness, speed up drying, and prevent the growth of molds, bacteria, and other microorganisms; however, excessive sulfur fumigation will alter the active ingredients of drugs and lead to excessive sulfur dioxide generation, which can have a negative effect on human health. With this in mind, hyperspectral technology can be used to detect sulfur dioxide residues in the near-infrared range in sulfur-fumigated Chuanbeimu and Chenpi specimens (He et al., 2017; Feng et al., 2019; Qiu Guangjun et al., 2024). In addition, hyperspectral technology can be used to monitor physical changes in Chinese herbal medicines during processing, such as changes in the moisture content and color, which directly affect the efficacy and storage stability of the final product (Bhargava et al., 2024). By analyzing the spectral data and known moisture contents of a large number of samples over different wavelength ranges, a model based on hyperspectral data and quality characteristics was established to achieve the rapid assessment and real-time monitoring of moisture, and to improve the final processing efficiency and product consistency (Xue et al., 2021).

#### 3.5.2 Quality assessment of processed products

Hyperspectral technology also has significant advantages for the quality assessment of processed Chinese herbal medicine products. Through the analysis of hyperspectral images, it is possible to identify Chinese herbal medicines that are contaminated by molds or insects. Notably, this approach overcomes the limitations associated with traditional methods, including a poor reproducibility, thereby providing accurate quality assessments for herbal medicines and improving their safety profiles (Zuo et al., 2023). For example, HSI has been used for the non-destructive detection of honeysuckle aphids and molds in jujubes, demonstrating a high accuracy and sensitivity (Wang et al., 2019a; Wu et al., 2013). In addition, when combined with machine learning algorithms, hyperspectral techniques have been reported to detect the impurities present in complex samples of processed herbal products (Manifold et al., 2021), and to assess the contents and distributions of their impurities. For example, researchers have fused hyperspectral and imaging data to successfully detect the adulteration of Ganoderma lucidum spore powder (Jiang et al., 2023a) and Panax ginseng powder (Zhang et al., 2022). Overall, the application of hyperspectral technology in the processing of Chinese herbal medicines not only improves the efficiency of quality control, but it also provides novel ideas and methods for compositional analysis.

## 4 Challenges and prospects

To sum up, HSI has the advantages of being non-destructive, fast, easy to operate, highly versatile, and providing multi-dimensional data. Compared to other techniques such as Near-Infrared Spectroscopy (NIR) and Raman Spectroscopy, HSI enables *in situ* analysis of Chinese medicinal materials preserving sample integrity and compositional distribution information, while simultaneously providing both spectral and spatial dimensional information, thus integrating a “data cube”. However, although HSI technology shows good application prospects in this field, previous research has mainly focused on simple identification and prediction protocols for single herbs or components in a laboratory environment. It is therefore necessary to develop large databases of compounds and herbs to promote the application of HSI technology on a large scale, and to ensure the safety and standardization of Chinese herbal medicines at the source (Liu et al., 2020).

**FIGURE 4:**
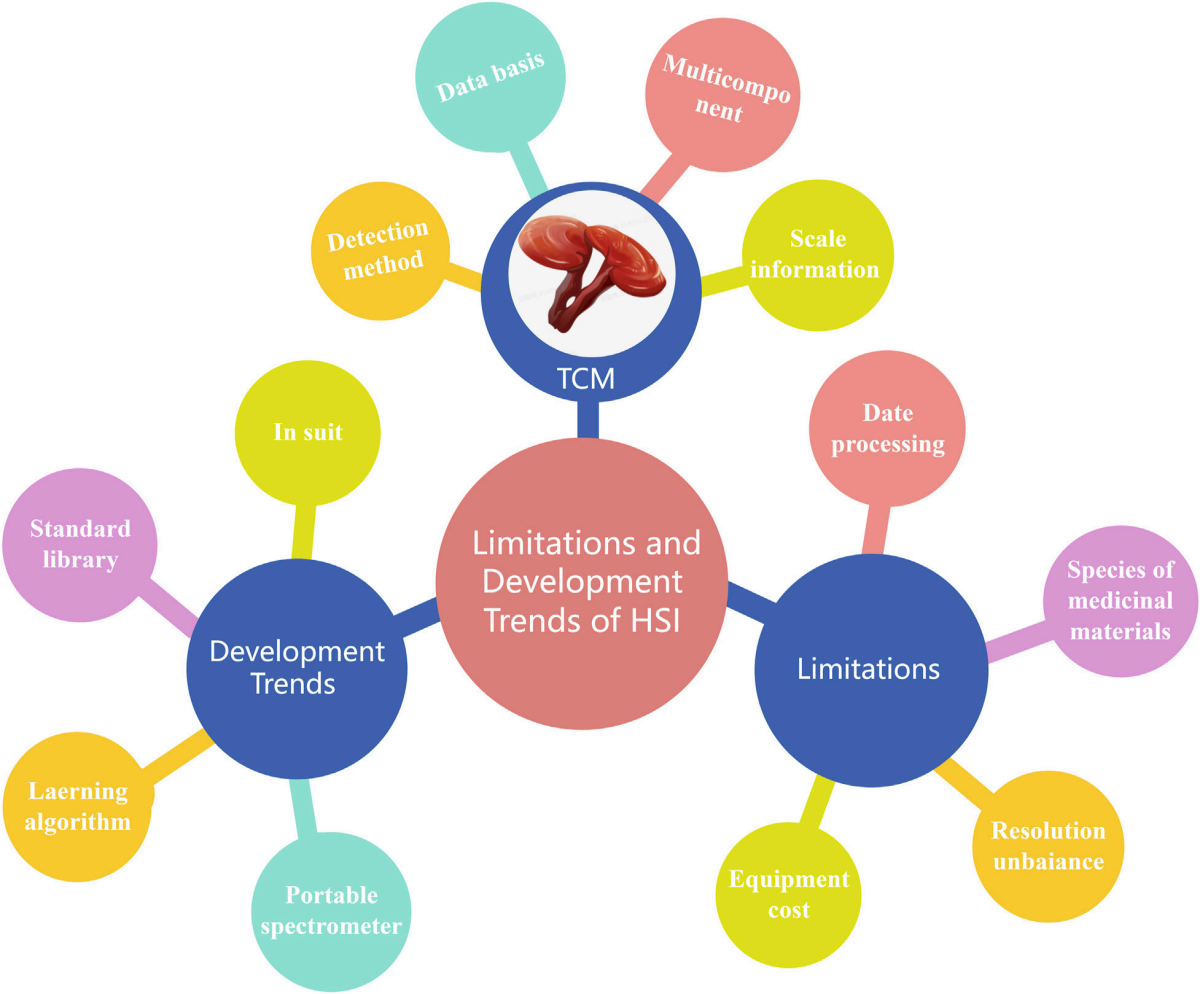
Limitations and trends associated with hyperspectral imaging systems.

A number of challenges are also associated with hyperspectral technology (Figure 4). Firstly, this approach is less integrated than other technologies because of the high data dimensions and computational complexity. In addition, the resulting complexity associated with data processing and feature extraction renders the selection and optimization of algorithms critical. Furthermore, in the context of practical applications, the huge number of species, origins, and processing methods associated with Chinese herbal medicines leads to wide-ranging spectral features. It is therefore difficult to establish a unified standard; this is unaided by imperfections in the spectral database and the testing process. Moreover, an imbalance remains between the spatial and spectral resolutions of hyperspectral sensors. Consequently, improving the spatial resolution while maintaining a high spectral resolution is an important research direction (Pan et al., 2024). Additionally, HSI equipment is expensive, and the costs associated with its related technologies are high. The spectral data processing and analysis protocols are also extremely time-consuming. Finally, future studies in this field should focus on reducing the costs associated with such innovative technologies, in addition to improving the real-time nature of spectral data acquisition and application to promote the widespread application of HSI. Recently, emerging technologies such as deep learning have been investigated to improve the processing efficiency and accuracy of spectral imaging data (Wang He et al., 2023).

## 5 Conclusion

This paper summarizes the application of HSI in the field of traditional Chinese medicinal materials, mainly focusing on species identification, origin classification, and component detection. With the analysis process as the framework, it elaborately describes the spectral data processing methods, texture information extraction methods, and data fusion strategies. It also analyzes a series of challenges faced by intelligent monitoring of traditional Chinese medicine from different perspectives. Given the significance of traditional Chinese medicinal materials in the medical field, it is suggested that future research in this area can further predict multiple chemical components and their mutual synergistic effects, promoting the transition of HSI from laboratory conditions to large-scale applications.

## References

[B1] Adesokan M. Otegbayo B. Oladeji Alamu E. Olutoyin M. A. Maziya-Dixon B. (2024). Evaluating the dry matter content of raw yams using hyperspectral imaging spectroscopy and machine learning. J. Food Compos. Analysis 135, 106692. 10.1016/j.jfca.2024.106692

[B2] Akimov M. U. Zhbanova E. V. Makarov V. N. Perova I. B. Shevyakova L. V. Vrzhesinskaya O. A. (2019). Nutrient value of fruit in promising strawberry varieties. Vopr. Pitan. 88, 64–72. 10.24411/0042-8833-2019-10019 31233690

[B3] Alibabaei S. Rahmani M. Tahmasbi M. Birgani M. J. T. Razmjoo S. (2023). Evaluating the gray level Co-occurrence matrix-based texture features of magnetic resonance images for glioblastoma multiform patients’ treatment response assessment. J. Med. Signals and Sensors 13, 261–271. 10.4103/jmss.jmss_50_22 PMC10559301 37809020

[B4] Arivazhagan S. Newlin Shebiah R. Ananthi S. Varthini S. V. (2013). Detection of unhealthy region of plant leaves and classification of plant leaf diseases using texture features. Agric. Eng. Int. CIGR J. 15, 211–217.

[B5] Badaró A. T. Garcia-Martin J. F. López-Barrera M. del C. Douglas F. B. Alvarez-Mateos P. (2020). Determination of pectin content in orange peels by near infrared hyperspectral imaging. Food Chem. 323, 126861. 10.1016/j.foodchem.2020.126861 32334320

[B6] Bai Y. Xiong Y. Huang J. Zhou J. Zhang B. (2019). Accurate prediction of soluble solid content of apples from multiple geographical regions by combining deep learning with spectral fingerprint features. Postharvest Biol. Technol. 156, 110943. 10.1016/j.postharvbio.2019.110943

[B7] Barnes E. M. Clarke T. R. Richards S. E. Colaizzi P. D. Haberland J. Kostrzewski M. (2000). “Coincident detection of crop water stress, nitrogen status and canopy density using ground based multispectral data,” in Proceedings of the fifth international conference on precision agriculture. Bloomington, MN, USA.

[B8] Barnes J. D. Balaguer L. Manrique E. Elvira S. Davison A. W. (1992). A reappraisal of the use of DMSO for the extraction and determination of chlorophylls a and b in lichens and higher plants. Environ. Exp. Bot. 32, 85–100. 10.1016/0098-8472(92)90034-y

[B9] Bhargava A. Sachdeva A. Sharma K. Alsharif M. H. Uthansakul P. Uthansakul M. (2024). Hyperspectral imaging and its applications: a review. Heliyon 10, e33208. 10.1016/j.heliyon.2024.e33208 39021975 PMC11253060

[B10] Broge N. H. Leblanc E. (2001). Comparing prediction power and stability of broadband and hyperspectral vegetation indices for estimation of green leaf area index and canopy chlorophyll density. Remote Sens. Environ. 76, 156–172. 10.1016/s0034-4257(00)00197-8

[B11] Cai J. Liu M. Qi Z. Shao Z. Zhou J. Guo Y. (2022). Renal cancer detection: fusing deep and texture features from histopathology images. BioMed Res. Int. 2022, 9821773. 10.1155/2022/9821773 35386304 PMC8979690

[B12] Cai Z. He M. Li C. Qi H. Bai R. Yang J. (2023a). Identification of chrysanthemum using hyperspectral imaging based on few-shot class incremental learning. Comput. Electron. Agric. 215, 108371. 10.1016/j.compag.2023.108371

[B13] Cai Z. Huang Z. He M. Li C. Qi H. Peng J. (2023b). Identification of geographical origins of Radix Paeoniae Alba using hyperspectral imaging with deep learning-based fusion approaches. Food Chem. 422, 136169. 10.1016/j.foodchem.2023.136169 37119596

[B14] Cao X. Zhang Y.-T. Wu Z.-F. Xu L.-F. Wang Z.-Q. Zeng H.-M. (2024). Scientific connotation of temperature control in processing of Chinese medicinal materials based on theory of quality evaluation through morphological identification. Zhongguo Zhong yao za zhi= Zhongguo Zhongyao Zazhi= China J. Chin. Materia Medica 49, 1196–1205. 10.19540/j.cnki.cjcmm.20231120.302 38621966

[B15] Chakraborty R. Rachdi I. Thiele S. Booysen R. Moritz K. Lorenz S. (2024). A spectral and spatial comparison of satellite-based hyperspectral data for geological mapping. Remote Sens. 16, 2089. 10.3390/rs16122089

[B16] Chen H. Qiao H. Lin B. Xu G. Tang G. Cai K. (2019). Study of modeling optimization for hyperspectral imaging quantitative determination of naringin content in pomelo peel. Comput. Electron. Agric. 157, 410–416. 10.1016/j.compag.2019.01.013

[B17] Chen J. M. (1996). Evaluation of vegetation indices and a modified simple ratio for boreal applications. Can. J. Remote Sens. 22, 229–242. 10.1080/07038992.1996.10855178

[B18] Chen Y. Jiang X. Liu Q. Wei Y. Wang F. Yan L. (2024). A hyperspectral imaging technique for rapid non-destructive detection of soluble solid content and firmness of wolfberry. J. Food Meas. Charact. 18, 7927–7941. 10.1007/s11694-024-02775-5

[B19] Chen Z.-Y. Wang L. Jiang C. Jin Y. Zhou J.-H. Nan T.-G. (2020). Rapid quality detection system of Lonicerae Japonicae Flos formula granules. Zhongguo Zhong yao za zhi= Zhongguo Zhongyao Zazhi= China J. Chin. Materia Medica 45, 1070–1075. 10.19540/j.cnki.cjcmm.20200112.103 32237448

[B20] Cheng R. Bai X. Guo J. Huang L. Zhao D. Liu Z. (2024). Hyperspectral discrimination of ginseng variety and age from Changbai Mountain area. Spectrochimica Acta Part A Mol. Biomol. Spectrosc. 307, 123613. 10.1016/j.saa.2023.123613 37976570

[B21] Cheshkova A. F. (2022). A review of hyperspectral image analysis techniques for plant disease detection and identif ication. Vavilov J. Genet. Breed. 26, 202–213. 10.18699/vjgb-22-25 PMC8983301 35434482

[B22] Christophe E. Léger D. Mailhes C. (2005). Quality criteria benchmark for hyperspectral imagery. IEEE Trans. Geoscience Remote Sens. 43, 2103–2114. 10.1109/tgrs.2005.853931

[B23] Colomer A. Igual J. Naranjo V. (2020). Detection of early signs of diabetic retinopathy based on textural and morphological information in fundus images. Sensors 20, 1005. 10.3390/s20041005 32069912 PMC7071097

[B24] Cruz-Tirado J. P. Lima Brasil Y. Lima A. F. Alva Pretel H. Teixeira Godoy H. Douglas B. (2023). Rapid and non-destructive cinnamon authentication by NIR-hyperspectral imaging and classification chemometrics tools. Spectrochimica Acta Part A Mol. Biomol. Spectrosc. 289, 122226. 10.1016/j.saa.2022.122226 36512964

[B25] Cui J. Li K. Jie H. Dong F. Wang S. Rodas-González A. (2022). Identification of near geographical origin of wolfberries by a combination of hyperspectral imaging and multi-task residual fully convolutional network. Foods 11, 1936. 10.3390/foods11131936 35804752 PMC9265825

[B26] Dai Y. Yan B. Xiong F. Bai R. Wang S. Guo L. (2024). Tanshinone content prediction and geographical origin classification of Salvia miltiorrhiza by combining hyperspectral imaging with chemometrics. Foods 13, 3673. 10.3390/foods13223673 39594089 PMC11593691

[B27] Dash J. Paul J. C. (2004). The MERIS terrestrial chlorophyll index. Int. J. Remote Sens. 25, 5403–5413. 10.1080/0143116042000274015

[B28] Daughtry C. S. T. Walthall C. L. Kim M. S. Brown De Colstoun E. McMurtrey J. E. Iii (2000). Estimating corn leaf chlorophyll concentration from leaf and canopy reflectance. Remote Sens. Environ. 74, 229–239. 10.1016/s0034-4257(00)00113-9

[B29] Ding R. Yu L. Wang C. Zhong S. Gu R. (2024). Quality assessment of traditional Chinese medicine based on data fusion combined with machine learning: a review. Crit. Rev. Anal. Chem. 54, 2618–2635. 10.1080/10408347.2023.2189477 36966435

[B30] Duan Z. Li H. Li C. Zhang J. Zhang D. Fan X. (2024). A CNN model for early detection of pepper Phytophthora blight using multispectral imaging, integrating spectral and textural information. Plant methods 20, 115. 10.1186/s13007-024-01239-7 39075512 PMC11288097

[B31] Farrar M. B. Wallace H. M. Brooks P. Yule C. M. Tahmasbian I. Dunn P. K. (2021). A performance evaluation of Vis/NIR hyperspectral imaging to predict curcumin concentration in fresh turmeric rhizomes. Remote Sens. 13, 1807. 10.3390/rs13091807

[B32] Feng L. Zhu S. Liu F. He Y. Bao Y. Zhang C. (2019). Hyperspectral imaging for seed quality and safety inspection: a review. Plant methods 15, 91–25. 10.1186/s13007-019-0476-y 31406499 PMC6686453

[B33] Gamon J. A. Penuelas J. Field C. B. (1992). A narrow-waveband spectral index that tracks diurnal changes in photosynthetic efficiency. Remote Sens. Environ. 41, 35–44. 10.1016/0034-4257(92)90059-s

[B34] Gao D. Zhang X. Zhou C. Fan W. Zeng T. Yang Q. (2021). MRI-aided kernel PET image reconstruction method based on texture features. Phys. Med. and Biol. 66, 15NT03. 10.1088/1361-6560/ac1024 34192685

[B35] Gao X. Dong W. Ying Z. Li G. Cheng Q. Zhao Z. (2024). Rapid discriminant analysis for the origin of specialty yam based on multispectral data fusion strategies. Food Chem. 460, 140737. 10.1016/j.foodchem.2024.140737 39116771

[B36] Gitelson A. A. (2004). Non-destructive assessment of chlorophyll carotenoid and anthocyanin content in higher plant leaves: principles and algorithms.

[B37] Gitelson A. A. Merzlyak M. N. (1997). Remote estimation of chlorophyll content in higher plant leaves. Int. J. remote Sens. 18, 2691–2697. 10.1080/014311697217558

[B38] Gu H. Wang S. Hu S. Wu X. Li Q. Zhang R. (2024). Identification of Panax notoginseng origin using terahertz precision spectroscopy and neural network algorithm. Talanta 274, 125968. 10.1016/j.talanta.2024.125968 38581849

[B39] Haboudane D. Miller J. R. Tremblay N. Zarco-Tejada P. J. Dextraze L. (2002). Integrated narrow-band vegetation indices for prediction of crop chlorophyll content for application to precision agriculture. Remote Sens. Environ. 81, 416–426. 10.1016/s0034-4257(02)00018-4

[B40] Hao J. Dong F. Li Y. Wang S. Cui J. Zhang Z. (2022a). Investigation of the data fusion of spectral and textural data from hyperspectral imaging for the near geographical origin discrimination of wolfberries using 2D-CNN algorithms. Infrared Phys. and Technol. 125, 104286. 10.1016/j.infrared.2022.104286

[B41] Hao J. Dong F. Wang S. Li Y. Cui J. Men J. (2022b). Combined hyperspectral imaging technology with 2D convolutional neural network for near geographical origins identification of wolfberry. J. Food Meas. Charact. 16, 4923–4933. 10.1007/s11694-022-01552-6

[B42] Hashemi-Nasab Parastar H. (2022). Vis-NIR hyperspectral imaging coupled with independent component analysis for saffron authentication. Food Chem. 393, 133450. 10.1016/j.foodchem.2022.133450 35751218

[B43] He J. Chen L. Chu B. Zhang C. (2018). Determination of total polysaccharides and total flavonoids in Chrysanthemum morifolium using near-infrared hyperspectral imaging and multivariate analysis. Molecules 23, 2395. 10.3390/molecules23092395 30235811 PMC6225252

[B44] He J. Zhang C. He Y. (2017). Application of near-infrared hyperspectral imaging to detect sulfur dioxide residual in the Fritillaria thunbergii bulbus treated by sulfur fumigation. Appl. Sci. 7, 77. 10.3390/app7010077

[B45] He J. Zhang C. Zhou L. He Y. (2021). Simultaneous determination of five micro-components in Chrysanthemum morifolium (Hangbaiju) using near-infrared hyperspectral imaging coupled with deep learning with wavelength selection. Infrared Phys. and Technol. 116, 103802. 10.1016/j.infrared.2021.103802

[B46] Heo S. Choi J.-Y. Kim J. Moon K.-D. (2021). Prediction of moisture content in steamed and dried purple sweet potato using hyperspectral imaging analysis. Food Sci. Biotechnol. 30, 783–791. 10.1007/s10068-021-00921-z 34249383 PMC8225792

[B47] Hu H. Wang T. Wei Y. Xu Z. Cao S. Fu L. (2023). Non-destructive prediction of isoflavone and starch by hyperspectral imaging and deep learning in Puerariae Thomsonii Radix. Front. Plant Sci. 14, 1271320. 10.3389/fpls.2023.1271320 37954990 PMC10634472

[B48] Huang C.-H. Chen W.-T. Chang Y.-C. Wu K.-T. (2024). An edge and trustworthy AI UAV system with self-adaptivity and hyperspectral imaging for air quality monitoring. IEEE Internet Things J. 11, 32572–32584. 10.1109/jiot.2024.3422470

[B49] Ibrahim A. Alghannam A. Eissa A. Firtha F. Kaszab T. Kovacs Z. (2021). Preliminary study for inspecting moisture content, dry matter content, and firmness parameters of two date cultivars using an NIR hyperspectral imaging system. Front. Bioeng. Biotechnol. 9, 720630. 10.3389/fbioe.2021.720630 34746101 PMC8570186

[B50] Indrayanto G. (2024). Regulation and standardization of herbal drugs: current status, limitation, challenge’s and future prospective. Profiles Drug Subst. Excipients Relat. Methodol. 49, 153–199. 10.1016/bs.podrm.2023.11.003 38423707

[B51] Jiang Z. Lv A. Zhong L. Yang J. Xu X. Li Y. (2023a). Rapid prediction of adulteration content in Atractylodis rhizoma based on data and image features fusions from near-infrared spectroscopy and hyperspectral imaging techniques. Foods 12, 2904. 10.3390/foods12152904 37569173 PMC10417609

[B52] Jiang Z. Zhong L. Xue J. Jiao L. Zhou F. Zhou Y. (2023b). Data fusion based on near-infrared spectroscopy and hyperspectral imaging technology for rapid adulteration detection of Ganoderma lucidum spore powder. Microchem. J. 193, 109190. 10.1016/j.microc.2023.109190

[B53] Kabir M. H. Guindo M. L. Chen R. Liu F. Luo X. Kong W. (2022). Deep learning combined with hyperspectral imaging technology for variety discrimination of Fritillaria thunbergii. Molecules 27, 6042. 10.3390/molecules27186042 36144775 PMC9501738

[B54] Kajino A. Bai W. Yoshimura N. Takayanagi M. (2021). Identification of peach and apricot kernels for traditional Chinese medicines using near-infrared spectroscopy. Vib. Spectrosc. 113, 103202. 10.1016/j.vibspec.2020.103202

[B55] Kiani S. Yazdanpanah H. Feizy J. (2023). 'Geographical origin differentiation and quality determination of saffron using a portable Hyperspectral imaging system. Infrared Phys. and Technol. 131, 104634. 10.1016/j.infrared.2023.104634

[B56] Kim M. S. Daughtry C. S. T. Chappelle E. W. McMurtrey J. E. Walthall C. L. (1994). “The use of high spectral resolution bands for estimating absorbed photosynthetically active radiation (A par),” in CNES, proceedings of 6th international symposium on physical measurements and signatures in remote sensing.

[B57] Lanjewar M. G. Panchbhai K. G. Patle L. B. (2024). Sugar detection in adulterated honey using hyper-spectral imaging with stacking generalization method. Food Chem. 450, 139322. 10.1016/j.foodchem.2024.139322 38613963

[B58] Li S. Song Q. Liu Y. Zeng T. Liu S. Jie D. (2023a). Hyperspectral imaging-based detection of soluble solids content of loquat from a small sample. Postharvest Biol. Technol. 204, 112454. 10.1016/j.postharvbio.2023.112454

[B59] Li S. Wang Y. Song H. Liu M. (2024a). Multi-spectral fusion and self-attention mechanisms for Gentiana origin identification via near-infrared spectroscopy. Chemom. Intelligent Laboratory Syst. 246, 105068. 10.1016/j.chemolab.2024.105068

[B60] Li X. Wei Z. Peng F. Liu J. Han G. (2023b). Non-destructive prediction and visualization of anthocyanin content in mulberry fruits using hyperspectral imaging. Front. Plant Sci. 14, 1137198. 10.3389/fpls.2023.1137198 37051079 PMC10083272

[B61] Li Y. Chen Z. Zhang F. Wei Z. Huang Y. Chen C. (2024b). Research on detection of potato varieties based on spectral imaging analytical algorithm. Spectrochimica Acta Part A Mol. Biomol. Spectrosc. 311, 123966. 10.1016/j.saa.2024.123966 38335591

[B62] Lin L. Zhang F. Zhang J. Ma C. Wang W. Lei L. (2021). Criticalquality attribute assessment of big brand traditional Chinese medicine: visualization method for quality control of Ginkgo Leaves Tablets based on spatial distribution uniformity. Zhongguo Zhong yao za zhi= Zhongguo Zhongyao Zazhi= China J. Chin. Materia Medica 46, 1616–1621. 10.19540/j.cnki.cjcmm.20210218.304 33982459

[B63] Liu B. Zhang H. Zhu J. Chen Y. Pan Y. Gong X. (2024a). Pixel-level recognition of trace mycotoxins in red ginseng based on hyperspectral imaging combined with 1DCNN-residual-BiLSTM-attention model. Sensors 24, 3457. 10.3390/s24113457 38894248 PMC11174722

[B64] Liu H. Bruning B. Garnett T. Berger B. (2020). The performances of hyperspectral sensors for proximal sensing of nitrogen levels in wheat. Sensors (Basel) 20, 4550. 10.3390/s20164550 32823800 PMC7472111

[B65] Liu H. Liu H. Li J. Wang Y. (2024b). ATR‐FTIR spectroscopy preprocessing technique selection for identification of geographical origins of gastrodia elata blume. J. Chemom. 38, e3579. 10.1002/cem.3579

[B66] Liu N. Townsend P. A. Naber M. R. Bethke P. C. Hills W. B. Wang Yi (2021). Hyperspectral imagery to monitor crop nutrient status within and across growing seasons. Remote Sens. Environ. 255, 112303. 10.1016/j.rse.2021.112303

[B67] Liu Q. Jiang X. Wang F. Zhu B. Yan L. Wei Y. (2024c). 'Detection of dried jujube from fresh jujube with different variety and maturity after hot air drying based on hyperspectral imaging technology. J. Food Compos. Analysis 133, 106378. 10.1016/j.jfca.2024.106378

[B68] Liu Y. Wang Q. Gao X. Xie A. (2019). Total phenolic content prediction in Flos Lonicerae using hyperspectral imaging combined with wavelengths selection methods. J. Food Process Eng. 42, e13224. 10.1111/jfpe.13224

[B69] Long W. Wang S.-R. Suo Y. Chen H. Bai X. Yang X. (2023). Fast and non-destructive discriminating the geographical origin of Hangbaiju by hyperspectral imaging combined with chemometrics. Spectrochimica Acta Part A Mol. Biomol. Spectrosc. 284, 121786. 10.1016/j.saa.2022.121786 36087403

[B70] Lu Y. Young S. Linder E. Whipker B. Suchoff D. (2022). 'Hyperspectral imaging with machine learning to differentiate cultivars, growth stages, flowers, and leaves of industrial hemp (Cannabis sativa L.). Front. Plant Sci. 12, 810113. 10.3389/fpls.2021.810113 35185960 PMC8847227

[B71] Luft L. Neumann C. Freude M. Blaum N. Jeltsch F. (2014). 'Hyperspectral modeling of ecological indicators–A new approach for monitoring former military training areas. Ecol. Indic. 46, 264–285. 10.1016/j.ecolind.2014.06.025

[B72] Lv Y. Dong F. Cui J. Jie H. Luo R. Wang S. (2023). Fusion of spectral and textural data of hyperspectral imaging for glycine content prediction in beef using SFCN algorithms. Food Anal. Methods 16, 413–425. 10.1007/s12161-022-02425-w

[B73] Ma J. Zhou X. Xie B. Wang C. Chen J. Zhu Y. (2023). Application for identifying the origin and predicting the physiologically active ingredient contents of gastrodia elata blume using visible–near-infrared spectroscopy combined with machine learning. Foods 12, 4061. 10.3390/foods12224061 38002117 PMC10670700

[B74] Manifold B. Men S. Hu R. Fu D. (2021). A versatile deep learning architecture for classification and label-free prediction of hyperspectral images. Nat. Mach. Intell. 3, 306–315. 10.1038/s42256-021-00309-y 34676358 PMC8528004

[B75] Merzlyak M. N. Gitelson A. A. Chivkunova O. B. Rakitin V. Y. U. (1999). Non‐destructive optical detection of pigment changes during leaf senescence and fruit ripening. Physiol. Plant. 106, 135–141. 10.1034/j.1399-3054.1999.106119.x

[B76] Metternicht G. (2003). 'Vegetation indices derived from high-resolution airborne videography for precision crop management. Int. J. remote Sens. 24, 2855–2877. 10.1080/01431160210163074

[B77] Nirere A. Sun J. Kama R. Vincent A. A. Nikubwimana F. D. Dusabe K. D. (2023). Nondestructive detection of adulterated wolfberry (*Lycium Chinense*) fruits based on hyperspectral imaging technology. J. Food Process Eng. 46, e14293. 10.1111/jfpe.14293

[B78] Pan S. Zhang X. Xu W. Yin J. Gu H. Yu X. (2022). Rapid On-site identification of geographical origin and storage age of tangerine peel by Near-infrared spectroscopy. Spectrochimica Acta Part A Mol. Biomol. Spectrosc. 271, 120936. 10.1016/j.saa.2022.120936 35121470

[B79] Pan Y. Zhang H. Chen Y. Gong X. Yan J. Zhang H. (2024). Applications of hyperspectral imaging technology combined with machine learning in quality control of traditional Chinese medicine from the perspective of artificial intelligence: a review. Crit. Rev. Anal. Chem. 54, 2850–2864. 10.1080/10408347.2023.2207652 37246728

[B80] Pechlivani E. M. Papadimitriou A. Pemas S. Ntinas G. Tzovaras D. (2023). 'IoT-based agro-toolbox for soil analysis and environmental monitoring. Micromachines 14, 1698. 10.3390/mi14091698 37763861 PMC10534498

[B81] Penuelas J. Frédéric B. Filella I. (1995). Semi-empirical indices to assess carotenoids/chlorophyll a ratio from leaf spectral reflectance. Photosynthetica 31, 221–230.

[B82] Ping J. Ying Z. Hao N. Miao P. Ye C. Liu C. (2024). Rapid and non-destructive identification of Panax ginseng origins using hyperspectral imaging, visible light imaging, and X-ray imaging combined with multi-source data fusion strategies. Food Res. Int. 192, 114758. 10.1016/j.foodres.2024.114758 39147491

[B83] Qiu G. Chen B. Lu H. Yue X. Deng X. Ouyang H. (2024a). Nondestructively determining soluble solids content of blueberries using reflection hyperspectral imaging technique. Agronomy 14, 2296. 10.3390/agronomy14102296

[B84] Qiu X. Dong Y. Jiang L. Fan W. Du G. Li P. (2024b). Portable near-infrared spectroscopy with variable selection-linear discriminant analysis technology for accurate and nondestructive detection of sulfur-fumigated Citri Reticulatae Pericarpium. LWT 205, 116518. 10.1016/j.lwt.2024.116518

[B85] Ran J. Xu H. Wang Z. Zhang W. Bai X. (2025). Non-destructive analysis of Ganoderma lucidum composition using hyperspectral imaging and machine learning. Front. Chem. 13, 1534216. 10.3389/fchem.2025.1534216 40078567 PMC11897918

[B86] Rondeaux G. Steven M. Baret F. (1996). 'Optimization of soil-adjusted vegetation indices. Remote Sens. Environ. 55, 95–107. 10.1016/0034-4257(95)00186-7

[B87] Roujean J.-L. Breon F.-M. (1995). 'Estimating PAR absorbed by vegetation from bidirectional reflectance measurements. Remote Sens. Environ. 51, 375–384. 10.1016/0034-4257(94)00114-3

[B88] Rouse Jr John W. Haas R. H. Deering D. W. Schell J. A. Harlan J. C. (1974). Monitoring the vernal advancement and retrogradation (green wave effect) of natural vegetation. In.

[B89] Samrat N. H. Johnson J. B. White S. Naiker M. Brown P. (2022). A rapid non-destructive hyperspectral imaging data model for the prediction of pungent constituents in dried ginger. Foods 11, 649. 10.3390/foods11050649 35267285 PMC8909893

[B90] Schmidt S. A. Ahn C. (2022). A protocol for digitizing colors: the case of measuring color variables for forested wetland soils. Environ. Monit. Assess. 194, 726. 10.1007/s10661-022-10420-1 36063235

[B91] Sharma S. R. Singh B. Kaur M. (2024). A hybrid encryption model for the hyperspectral images: application to hyperspectral medical images. Multimedia Tools Appl. 83, 11717–11743. 10.1007/s11042-023-15587-4

[B92] Shen D.-P. Jiang P. Zhan C.-S. (2024). Quality control of Tianwang Buxin Pills based on UPLC fingerprint and multi-component quantification. Zhongguo Zhong yao za zhi= Zhongguo Zhongyao Zazhi= China J. Chin. Materia Medica 49, 1240–1248. 10.19540/j.cnki.cjcmm.20231123.301 38621970

[B93] Shi L. Li L. Zhang F. Lin Y. (2022). Nondestructive detection of Panax notoginseng saponins by using hyperspectral imaging. Int. J. Food Sci. and Technol. 57, 4537–4546. 10.1111/ijfs.15790

[B94] Shi X. Lin X. Lei Y. Wu J. Lv X. Zhou Y. (2024). A study on pigment composition of buddhist cave paintings based on hyperspectral technology. Materials 17, 5147. 10.3390/ma17215147 39517424 PMC11547554

[B95] Sun J. Yao K. Cheng J. Xu M. Zhou X. (2024). Nondestructive detection of saponin content in Panax notoginseng powder based on hyperspectral imaging. J. Pharm. Biomed. Analysis 242, 116015. 10.1016/j.jpba.2024.116015 38364344

[B96] Sun X. Zhang D.-T. Wang H. Zhou C. Yang J. Peng D.-Y. (2023). Origin identification of Poria cocos based on hyperspectral imaging technology. Zhongguo Zhong yao za zhi= Zhongguo Zhongyao Zazhi= China J. Chin. Materia Medica 48, 4337–4346. 10.19540/j.cnki.cjcmm.20230512.102 37802860

[B97] Tang N. Sun J. Yao K. Zhou X. Tian Y. Cao Y. (2021). Identification of *Lycium barbarum* varieties based on hyperspectral imaging technique and competitive adaptive reweighted sampling‐whale optimization algorithm‐support vector machine. J. Food Process Eng. 44, e13603. 10.1111/jfpe.13603

[B98] Torres-Cobos B. Tres A. Vichi S. Guardiola F. Rovira M. Romero A. (2025). Comparative analysis of spectroscopic methods for rapid authentication of hazelnut cultivar and origin. Spectrochimica Acta Part A Mol. Biomol. Spectrosc. 326, 125367. 10.1016/j.saa.2024.125367 39531898

[B99] Vigier B. J. Pattey E. Strachan I. B. (2004). Narrowband vegetation indexes and detection of disease damage in soybeans. IEEE Geoscience Remote Sens. Lett. 1, 255–259. 10.1109/lgrs.2004.833776

[B100] Wang C. Wang S. He X. Wu L. Li Y. Guo J. (2020). Combination of spectra and texture data of hyperspectral imaging for prediction and visualization of palmitic acid and oleic acid contents in lamb meat. Meat Sci. 169, 108194. 10.1016/j.meatsci.2020.108194 32521405

[B101] Wang H. Liu S. Li W. Lyu M. (2023a). Research and application of intelligent hyperspectral analysis technology for Chinese materia medica. Zhongguo Zhong yao za zhi= Zhongguo Zhongyao Zazhi= China J. Chin. Materia Medica 48, 4320–4327. 10.19540/j.cnki.cjcmm.20230512.104 37802858

[B102] Wang M. Yao P. Sun P. Wen L. Chen X.-J. (2022). 'Key quality factors for Chinese herbal medicines entering the EU market. Chin. Med. 17, 29. 10.1186/s13020-022-00583-x 35193628 PMC8861989

[B103] Wang Q. Liu Y. Gao X. Xie A. Yu H. (2019a). Potential of hyperspectral imaging for nondestructive determination of chlorogenic acid content in Flos Lonicerae. J. Food Meas. Charact. 13, 2603–2612. 10.1007/s11694-019-00180-x

[B104] Wang Q. Liu Y. Xu Q. Feng J. Yu H. (2019b). Identification of mildew degrees in honeysuckle using hyperspectral imaging combined with variable selection. J. Food Meas. Charact. 13, 2157–2166. 10.1007/s11694-019-00136-1

[B105] Wang Q. Zhang Y. Yang B. (2023b). Development status of novel spectral imaging techniques and application to traditional Chinese medicine. J. Pharm. Analysis 13, 1269–1280. 10.1016/j.jpha.2023.07.007 PMC10759257 38174122

[B106] Wang Y. Xiong F. Zhang Y. Wang S. Yuan Y. Lu C. (2023c). Application of hyperspectral imaging assisted with integrated deep learning approaches in identifying geographical origins and predicting nutrient contents of Coix seeds. Food Chem. 404, 134503. 10.1016/j.foodchem.2022.134503 36219966

[B107] Wang Y. Zou B. Chai L. Lin Z. Feng H. Tang Y. (2024a). Monitoring of soil heavy metals based on hyperspectral remote sensing: a review. Earth-Science Rev. 254, 104814. 10.1016/j.earscirev.2024.104814

[B108] Wang Z. Yin Y. Yu H. Yuan Y. (2024b). A LIBSVM quality assessment model for apple spoilage during storage based on hyperspectral data. Anal. Methods 16, 4765–4774. 10.1039/d4ay00678j 38958385

[B109] Wei X. Huang L. Li S. Gao S. Jie D. Guo Z. (2023). Fast determination of amylose content in Lotus seeds based on hyperspectral imaging. Agronomy 13, 2104. 10.3390/agronomy13082104

[B110] Wei Y. Liu Q. Fan S. Jiang X. Chen Y. Wang F. (2024b). 'Development of a predictive model for assessing quality of winter jujube during storage utilizing hyperspectral imaging technology. J. Food Process Eng. 47, e14688. 10.1111/jfpe.14688

[B111] Wei Y. Yuan M. Hu H. Xu H. Mao X. (2024a). Estimation for soluble solid content in Hetian jujube using hyperspectral imaging with fused spectral and textural Features. J. Food Compos. Analysis 128, 106079. 10.1016/j.jfca.2024.106079

[B112] Wu B. Zhang M. Zeng H. Tian F. Potgieter A. B. Qin X. (2023). Challenges and opportunities in remote sensing-based crop monitoring: a review. Natl. Sci. Rev. 10, nwac290. 10.1093/nsr/nwac290 36960224 PMC10029851

[B113] Wu L. He J. Liu G. He X. Wang W. Wang S. (2013). Non-destructive detection of insect hole in jujube based on near-infrared hyperspectral imaging. Chin. J. Luminescence 34, 1527–1532. 10.3788/fgxb20133411.1527

[B114] Wu N. Zhang C. Bai X. Du X. He Y. (2018). Discrimination of chrysanthemum varieties using hyperspectral imaging combined with a deep convolutional neural network. Molecules 23, 2831. 10.3390/molecules23112831 30384477 PMC6278476

[B115] Xiao Q. Bai X. Gao P. He Y. (2020). Application of convolutional neural network-based feature extraction and data fusion for geographical origin identification of radix astragali by visible/short-wave near-infrared and near infrared hyperspectral imaging. Sensors 20, 4940. 10.3390/s20174940 32882807 PMC7506783

[B116] Xu M. Dai J. Zhang G. Hou W. Mu Z. Chen P. (2024). 'Inversion of Glycyrrhiza chlorophyll content based on hyperspectral imagery. Agronomy 14, 1163. 10.3390/agronomy14061163

[B117] Xu Q. Yin Y. Liu Y. Ma Q. Chen X. Zhao J. (2023). Simultaneous determination of six α-dicarbonyl compounds in traditional Chinese medicines using high‐performance liquid chromatography‐fluorescence detector with pre‐column derivatization. J. Sep. Sci. 46, 2300435. 10.1002/jssc.202300435 37548124

[B118] Xu Y. Liu L. Huang M. Ning X. (2019). High accuracy determination of Angelica dahurica origin based on near infrared spectroscopy and a random forest pruning algorithm. J. Near Infrared Spectrosc. 27, 278–285. 10.1177/0967033519841127

[B119] Xue Q. Qi M. Li Z. Yang B. Li W. Wang F. (2021). Fluorescence hyperspectral imaging system for analysis and visualization of oil sample composition and thickness. Appl. Opt. 60, 8349–8359. 10.1364/ao.432851 34612932

[B120] Yang C. Ma X. Guan H. Li L. Fan B. (2022). A rapid recognition method of Auricularia auricula varieties based on near-infrared spectral characteristics. Infrared Phys. and Technol. 125, 104239. 10.1016/j.infrared.2022.104239

[B121] Yang L. Xia Y. Zhang M. Zhu M. Feng L. Jia X. (2020). Practice and development of structural characteristics and quality control of multi-dimensional structure of basic components of genuine material of Moutan Cortex. Zhongguo Zhong yao za zhi= Zhongguo Zhongyao Zazhi= China J. Chin. Materia Medica 45, 3340–3350. 10.19540/j.cnki.cjcmm.20200622.301 32726049

[B122] Yao L. Wang Q. Yang J. Zhang Y. Zhu Y. Cao W. (2019). UAV-borne dual-band sensor method for monitoring physiological crop status. Sensors 19, 816. 10.3390/s19040816 30781552 PMC6412810

[B123] Yi T. Lin C. En-Ci J. Ji-Zhong Y. (2020). Application and prospects of hyperspectral imaging and deep learning in traditional Chinese medicine in context of AI and industry 4.0', Zhongguo Zhong yao za zhi= Zhongguo Zhongyao. Zazhi= China J. Chin. Materia Medica 45, 5438–5442. 10.19540/j.cnki.cjcmm.20200630.603 33350203

[B124] Yu D.-X. Guo S. Yang J. Yan H. Zhang Z. Duan J. (2022). Application and prospect of stable isotope technology in tracing geographical origin of Chinese herbal medicines. Zhongguo Zhong yao za zhi= Zhongguo Zhongyao Zazhi= China J. Chin. Materia Medica 47, 862–871. 10.19540/j.cnki.cjcmm.20211105.102 35285184

[B125] Zarco-Tejada P. J. Pushnik J. C. Dobrowski S. Ustin S. L. (2003). Steady-state chlorophyll a fluorescence detection from canopy derivative reflectance and double-peak red-edge effects. Remote Sens. Environ. 84, 283–294. 10.1016/s0034-4257(02)00113-x

[B126] Zhang C. Wu W. Zhou L. Cheng H. Ye X. He Y. (2020). Developing deep learning based regression approaches for determination of chemical compositions in dry black goji berries (Lycium ruthenicum Murr.) using near-infrared hyperspectral imaging. Food Chem. 319, 126536. 10.1016/j.foodchem.2020.126536 32146292

[B127] Zhang D. Yang J. Cheng M. Wang H. Peng D. Zhang X. (2023a). Origin identification of Polygonatum cyrtonema based on hyperspectral data. Zhongguo Zhong yao za zhi= Zhongguo Zhongyao Zazhi= China J. Chin. Materia Medica 48, 4347–4361. 10.19540/j.cnki.cjcmm.20230512.103 37802861

[B128] Zhang F. Shi L. Li L. Zhou Y. Tian L. Cui X. (2022). Nondestructive detection for adulteration of panax notoginseng powder based on hyperspectral imaging combined with arithmetic optimization algorithm‐support vector regression. J. Food Process Eng. 45, e14096. 10.1111/jfpe.14096

[B129] Zhang F. Lin L. Zeng J. Zhu M. Lu Y. Zhang H. (2021). Critical quality attribute assessment of big brand traditional Chinese medicine: visualization of blending process for rare medicines in Tongren Niuhuang Qingxin Pills based on spatial distribution uniformity. China J. Chin. Materia Medica 46, 1585–1591. 10.19540/j.cnki.cjcmm.20210218.303 33982455

[B130] Zhang G. Abdulla W. (2022). New Zealand honey botanical origin classification with hyperspectral imaging. J. Food Compos. Analysis 109, 104511. 10.1016/j.jfca.2022.104511

[B131] Zhang H. Pan Y. X. Chen Y. Zhang H. Xie J. H. Gong X. C. (2024a). Improving the geographical origin classification of Radix glycyrrhizae (licorice) through hyperspectral imaging assisted by U-Net fine structure recognition. Analyst 149, 1837–1848. 10.1039/d3an02064a 38345564

[B132] Zhang L. Guan Y. Wang N. Ge F. Zhang Y. Zhao Y. (2023b). Identification of growth years for Puerariae Thomsonii Radix based on hyperspectral imaging technology and deep learning algorithm. Sci. Rep. 13, 14286. 10.1038/s41598-023-40863-6 37653027 PMC10471754

[B133] Zhang W. Bai X. Guo J. Yang J. Yu B. Chen J. (2024b). Hyperspectral imaging for *in situ* visual assessment of Industrial-Scale ginseng. Spectrochim. Acta A Mol. Biomol. Spectrosc. 321, 124700. 10.1016/j.saa.2024.124700 38925038

[B134] Zhang Y. Li Y. Zhou C. Zhou J. Nan T. Yang J. (2023c). Rapid and nondestructive identification of origin and index component contents of Tiegun yam based on hyperspectral imaging and Chemometric method. J. Food Qual. 2023, 1–11. 10.1155/2023/6104038

[B135] Zhang Y. Liu J. (2025). Non‐destructive detection of milk nutritional components based on hyperspectral imaging. J. Food Sci. 90, e17621. 10.1111/1750-3841.17621 39731729

[B136] Zhang Y. Ma L. Yi W. Wu L. (2024c). Research on the adulteration of Lycium barbarum based on hyperspectral imaging technology combined with deep learning algorithm. J. Food Compos. Analysis 136, 106765. 10.1016/j.jfca.2024.106765

[B137] Zhao Q. Miao P. Liu C. Yang Y. Zheng L. (2024). Accurate and non-destructive identification of origins for lily using near-infrared hyperspectral imaging combined with machine learning. J. Food Compos. Analysis 129, 106080. 10.1016/j.jfca.2024.106080

[B138] Zhao W. Ma F. Yu H. Li Z. (2023). Inversion model of salt content in alfalfa-covered soil based on a combination of UAV spectral and texture information. Agriculture 13, 1530. 10.3390/agriculture13081530

[B139] Zhou C. Gong Y. Fang S. Yang K. Peng Y. Wu X. (2022a). Combining spectral and wavelet texture features for unmanned aerial vehicles remote estimation of rice leaf area index. Front. Plant Sci. 13, 957870. 10.3389/fpls.2022.957870 35991436 PMC9386364

[B140] Zhou C. Wang H. Yang J. Zhang X. (2022b). Origin identification of Gardeniae Fructus based on hyperspectral imaging technology. Zhongguo Zhong yao za zhi= Zhongguo Zhongyao Zazhi= China J. Chin. Materia Medica 47, 6027–6033. 10.19540/j.cnki.cjcmm.20220809.103 36471926

[B141] Zhou Y. Zuo Z. Xu F. Wang Y. (2020). Origin identification of Panax notoginseng by multi-sensor information fusion strategy of infrared spectra combined with random forest. Spectrochimica Acta Part A Mol. Biomol. Spectrosc. 226, 117619. 10.1016/j.saa.2019.117619 31606667

[B142] Zuo J. Peng Y. Li Y. Zou W. Chen Y. Huo D. (2023). Nondestructive detection of nutritional parameters of pork based on NIR hyperspectral imaging technique. Meat Sci. 202, 109204. 10.1016/j.meatsci.2023.109204 37146500

